# Food Plants and Environmental Contamination: An Update

**DOI:** 10.3390/toxics12050365

**Published:** 2024-05-15

**Authors:** Nicoletta Guerrieri, Stefania Mazzini, Gigliola Borgonovo

**Affiliations:** 1 National Research Council, Water Research Institute, Largo Tonolli 50, I-28922 Verbania, Italy; 2 DeFENS Department of Food, Environmental and Nutritional Sciences, via Celoria 2, I-20133 Milano, Italy; stefania.mazzini@unimi.it (S.M.);

**Keywords:** heavy metals, POPs, pesticides, emerging contaminants, food safety

## Abstract

Food plants are the basis of human nutrition, but, in contaminated places, they can uptake contaminants. Environmental contamination and climate change can modify food quality; generally, they have a negative impact on and imply risks to human health. Heavy metals, like lead, arsenic, cadmium, and chromium, can be present at various environmental levels (soil, water, and atmosphere), and they are widely distributed in the world. Food plants can carry out heavy metal bioaccumulation, a defense pathway for plants, which is different for every plant species. Accumulation is frequent in the roots and the leaves, and heavy metals can be present in fruits and seeds; As and Cd are always present. In addition, other contaminants can bioaccumulate in food plants, including emerging contaminants, like persistent organic pollutants (POPs), pesticides, and microplastics. In food plants, these are present in the roots but also in the leaves and fruits, depending on their chemical structure. The literature published in recent years was examined to understand the distribution of contaminants among food plants. In the literature, old agronomical practices and new integrated technology to clean the water, control the soil, and monitor the crops have been proposed to mitigate contamination and produce high food quality and high food safety.

## 1. Introduction

In past years, the growth of food crop cultivation has become strictly linked to productivity through the extensive use of soil, water, fertilizers, and pesticides. In this context, ensuring food safety has been crucial to stop such behavior. Intensive agricultural methods have resulted in soil erosion and diminished soil fertility, primarily due to the spread of numerous fertilizers and pesticides into the soil and water systems. Improper waste management practices, coupled with the depletion of local water resources, have resulted in environmental contamination and bioaccumulation in food plants. Good agronomic practices can change the soil’s richness, thus improving microorganisms and invertebrates and modifying the soil’s texture and food plant productivity [1]. Agricultural products should involve a low carbon footprint and preserve clean and safe waters around the globe [2]. Climate changes and their impact on agriculture require new approaches to food production, implementing sustainable agricultural practices, precision agriculture, pest management, and food security. The plant productivity can be improved by decreasing the number of pesticides used and by looking for new varieties resistant to hydric stress, high temperature, and salinity. Industrial emissions and human activities produce airborne contaminants, particulate matter, heavy metals, and volatile organic compounds, which can deposit on vegetal crop surfaces, waterways, and soil, thus increasing the contamination. Food plants are natural systems in equilibrium with the air, water, and soil, and their quality affects the plant’s production. An important challenge for the future will be the transformation of agriculture through technology and innovation into a circular economy system [1]. The FAO promotes a One Health approach as part of the agrifood system’s transformation for the health of people, animals, plants, and the environment. The One Health approach will be necessary to prevent, detect, and control diseases, enhance food safety, and achieve the Sustainable Development Goals (SDGs). The environmental sustainability of the globe shows that there is a general deterioration situation that needs to be changed [3]. The literature published in recent years was examined to understand the distribution of contaminants among food plants and new insights from the research to measure and reduce environmental contaminants.

## 2. Contamination and Vegetal Crop Systems

Bioaccumulation of toxic contaminants by vegetal crops has become a major challenge for farmers and plant breeders, as well as consumers. Air, soil, and water pollution contribute to the presence of heavy metals, persistent organic pollutants (POPs), naturally occurring toxins, and other pollutants in the ecosystem. These contaminations are mainly associated with anthropogenic activity. The toxicity of POPs manifests itself over a long range of action from the emitting source in the environment [4]; the concentrations of these compounds have triggered considerable worry regarding the ecosystem over the last few years, thus increasing monitoring programs and sanctions in many states.

Plants have efficient defense systems against many pollutants, allowing them to live and develop even in contaminated areas [5]. The combination of different metal behaviors affects plants’ bioaccumulation; the metals in the soil cannot be degraded, and they often have a long residence time. Furthermore, the plants can uptake metals with chemical–physical similarities to essential ions [6]. The rhizosphere found in the soil around the roots increases the efficiency of the process and the survival of the plants [7]. The metals can change their oxidation state, often through microorganisms, or they can be complexed with other molecules, which slows down their adsorption and bioaccumulation in the plants. These molecules are known as soil improvers. Plants can accumulate metals, microparticles, and various molecules within their roots and then transport and distribute them through the stem, ultimately depositing them in the leaves, flowers, and fruits along a natural elimination pathway.

**Figure 1:**
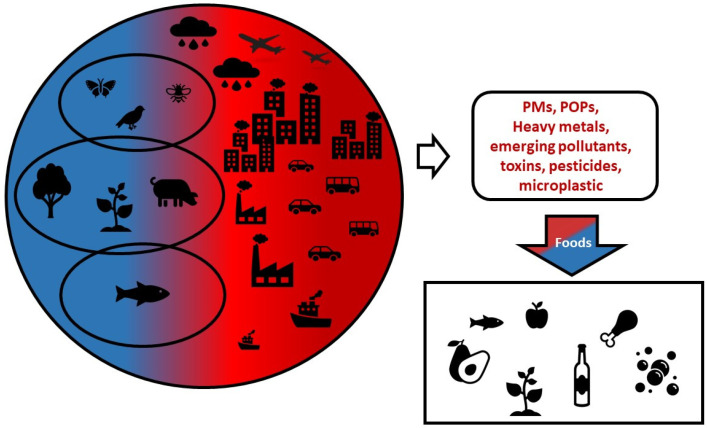
Relationship between anthropogenic environmental contaminants and foods. Blue: no contaminants. Red: anthropogenic contaminants.

The uptake of contaminants (Figure 1) by edible plants through absorption and subsequent accumulation along the food chain is a potential threat to animal and human health. Several factors influence the uptake of contaminants by plants, including the temperature, pH, salinity, nutrients, organic matter availability, and rhizosphere. Low pH and high salinity increase the availability, mobility, and redistribution in the soil [8]. Several studies have examined the accumulation of heavy metals depending on the plant species, while the absorbing efficiency is measured through transfer factors and the bioaccumulation index [9,10,11]. The consumption of contaminated food can contribute to unwanted effects on human health.

### 2.1. Heavy Metal Contamination

Environmental heavy metal contamination, which arises from industrial growth and anthropogenic activities, is one of the major environmental health challenges, and it is dangerous for bioaccumulation through the food chain. Heavy metals (HMs) of different sources spread into the environment through the soil, water, and air; in particular, HMs of anthropogenic activities are dangerous because of their instability and solubility, which leads to a high bioavailability [12].

HMs are defined as any metallic element having an atomic number greater than 20 and high density. The term applies to the group of metals with an atomic density greater than 5 g/cm^3^. HMs are toxic even at ppb levels. The most widespread are lead (Pb), cadmium (Cd), arsenic (As), and chromium (Cr). In the latest Codex Alimentarius [13], Pb, Cd, and As limits are listed for different foods. Cd bioaccumulation by cocoa trees (*Theobroma cacao* L.) sometimes reaches unacceptably high levels in cocoa beans [14], and good agronomical practices have been suggested to reduce its bioaccumulation. Both Cd and As are widely included in the list prepared by the International Agency for Research on Cancer (IARC) as class 1 human carcinogens. The toxicity at high levels of HMs is usually associated with metabolic disorders in plants and a reduced ability to assimilate nutrients, evident chlorosis, growth inhibition, and low biomass output. In laboratory experiments, *Ocimum Basilicum* growth on highly contaminated soil from mining activity showed accumulation in the leaves of Cd, Cr, and Pb. At lower levels of HMs, *Ocimum Basilicum* can activate the defense metabolic pathways that drive the plants to grow with a stimulation effect. The HMs accumulate in the roots and not in the leaves, and the plants present high biomass output. The plant’s metal uptake depends on many biotic and abiotic factors and chemical characteristics of metals that promote metal competition or antagonism [6].

Lettuce is a potential bioaccumulator of Pb, as reported in the literature [15]. This vegetable is one of the most widely utilized worldwide for its beneficial health properties, and a better knowledge of the sequestration mechanisms is recommended. Often, more than one metal enrichment has been observed, rather than just a single occurrence, with complex chemistry competition. Pb, As, and Cd were present in lettuce crops in Argentina [16]; the compost amendments improved soil fertility and reduced the Pb availability and toxicological risk, but it will be necessary to improve agronomic practices in soils with higher HM contamination in order to produce lettuce without toxicological risks. Coriander can bioaccumulate As and Cr, spinach can bioaccumulate As, Cr, and Cd, and cabbage bioaccumulates less Cr and As than spinach [17]. In spinach, the mixture of Cd and Pb increases the toxicity and the metals’ uptake, changing the metabolic parameters [18].

Fruits, seeds, and flowers in plants are possible final deposits of detoxification pathways. Tomatoes grown in soils from rural areas in Romania moderately contaminated by Cd and poorly contaminated with Pb and Cr showed low bioaccumulation and a low bioconcentration factor, indicating the necessity of monitoring the crop and the soil [19]. Sometimes, a higher level of metals was measured in the crop in a moderately contaminated Cd field [14]. HM accumulation in tomato showed higher values of Cr, As, Cd, and Pb; cauliflower showed higher values of Cr and As; and round gourd only showed As [6]. Wastewater irrigation is an agricultural practice that is widespread in the world; it is an important source of HMs, which can bioaccumulate in plants [9,11]. In the last few years, wastewater and water contamination was investigated in tomatoes, which accumulated Pb and Cd [8]; in spinach, which accumulated Pb [12]; in the roots of turnip and leek, which accumulated As; in black cabbage, which accumulated As in the leaves; and in tomato, which accumulated As only in the root [20].

In vegetable roots, tubers, and bulbs, the enrichment of As was always present in turnip, radish, potato, onion, and garlic; Cr was present in lower values; and Cd was only present in turnip [17]. Onions can uptake As, but it is located in the outer shell [21]. Carrots can uptake high levels of Pb [22], and bio-inoculation (a microbial consortium of *Pseudomonas putida* and *Pseudomonas aeruginosa*) can reduce the uptake of Pb efficiently from the rhizosphere. Carrots can uptake As from contaminated soils; the metal is mainly located in the upper part of the carrot. All carrot varieties, including orange, black, red, and white, can bioaccumulate the metal [23]. In potato, passivation (sepiolite, calcium magnesium phosphate) management applied to contaminated soil improved the soil health (higher soil enzymatic activity) and reduced the accumulation of Cd, Pb, and As [24].

A study on two different cultivars of soybean (*Glycine max* L.) [25] and Cr uptake was performed in hydroponic experiments with different conditions and included an analysis of the oxidative damage, the antioxidant enzymatic and non-enzymatic response, and the expression levels of genes. The two cultivars showed different responses to Cr addition, sensible or tolerant, with different pathway responses. The roots contained a high amount of Cr, but only a small quantity was translocated to the shoots and leaves. The brassinosteroids foliar application reduced the Cr toxicity and uptake, and it should be utilized in contaminated areas [25].

In hydroponic experiments, quinoa plants (*Chenopodium Quinoa* Willd) were contaminated with Cd and Pb, and the plants were always stressed. The plants’ responses to the single metal contamination and the mixture of the two metals were different, as the two metals led to additive negative effects on the plant growth and accumulation of Cd in the root [26].

Rice can bioaccumulate high amounts of Cd. In China, this is a serious threat to public health, as the contamination is on a large scale, and phytoremediation is an ideal strategy for Chinese farmlands. A study explored the agronomical practice of rotation between chicory and rice. The crop rotation did not affect the rice yields, the Cd uptake in rice decreased every year, and, after the fourth year, all of the varieties, including the low-Cd and high-Cd varieties, were close to the safety standards. The soil also increased its texture and nutrient richness [27]. As it can be accumulated in rice to a high level, As is widespread in China, Asia, and, often, in Europe, and studies on innovative methods to reduce its uptake have been carried out. Glutamic acid in foliar application on aromatic rice enhanced the antioxidant mechanism, thus regulating the amino acid metabolites’ profiles and reducing As toxicity to a safe level in the plants’ early growth stage [28,29]. Soil treatment with silica nanoparticles contributes to the mitigation of metal toxicity in cereals and pulses. In rice, it reduces the uptake of Pb, Cd, Cr, and As; in wheat, it reduces the uptake of Cd; in maize, it reduces the uptake of As; in pea, it reduces the uptake of Cr; and, in bean, it reduces the uptake of Pb and Cd [30].

Wild fruits (raspberry, blackberry) can accumulate high levels of As [31]. Grapefruit and kinnow fruit grown on soil contaminated by wastewater accumulated Pb and Cd [32].

**Figure 2:**
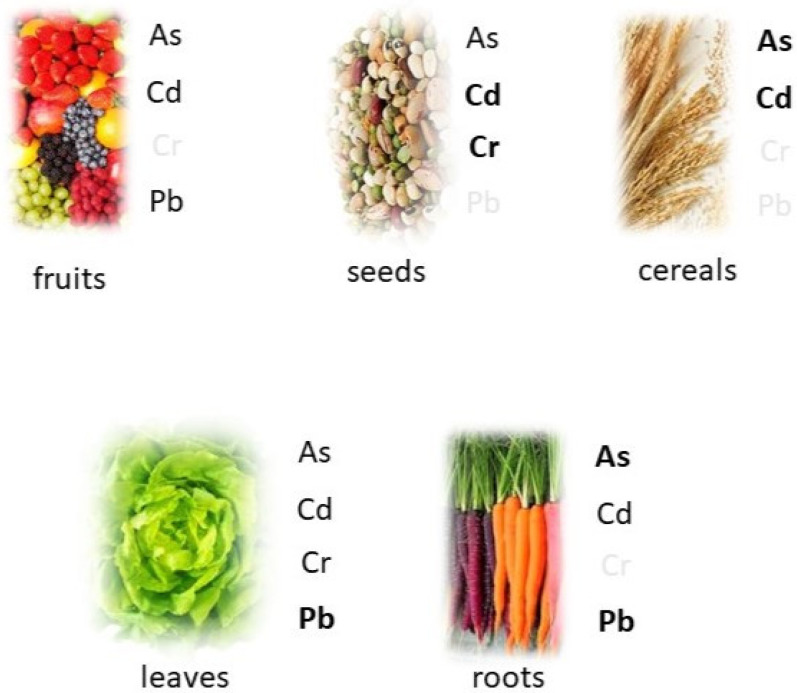
Metal distribution in food plants: fruits, seeds, cereals, leaves, and roots.

In summary, the HMs can be found in different vegetables. The bulbs and roots (onions, carrots, and potatoes) can always accumulate As, but it is often located in the external or upper part of the vegetable. Sometimes, Pb is also present. Leaves (lettuce, spinach, black cabbage, basil, and coriander) can accumulate Pb, As, Cr, and Cd, but the translocation from the roots to the leaves depends on the microorganism presence in the soil, which can change its safety value. The literature about tomatoes is heterogeneous. Pb and Cd can accumulate in the fruit, and As can accumulate in the root. Among cereals, rice can uptake As and Cd, but the uptake could be reduced with new agricultural treatments. Also, wheat and maize can accumulate Cd and As, respectively. Quinoa can accumulate Cd and Pb, but mainly in the roots. Soybeans accumulate Cr in the roots, but it can be reduced with cultivar selection and agronomical practices. Cocoa trees can accumulate Cd in the seeds, but good agronomical practices can reduce the uptake. Wild fruits can accumulate As, and grapefruit and kinnow can accumulate Pb and Cd; also, in this case, the metal accumulation can be reduced with cultivar selection. A graphic representation is shown in Figure 2. Metal control and monitoring should be a routine practice along with the analysis of soil texture and nutrient richness to optimize the cultivar selection to produce healthy and safe foods.

### 2.2. POPs and Emerging Contaminants

POPs are organic substances that persist in the environment and accumulate in living organisms; they are a risk to the environment and our health [33]. The semi-volatile properties and high photostability of many POPs lead to long-range transport and resilience in the atmosphere before then falling back into the waters and soil. They can be transported by air and water across international borders, reaching regions where they have never been produced or used [4]. POPs are regulated worldwide by the Stockholm Convention [34] and the Aarhus Protocol [35]. These international treaties are implemented in the European Union through the POPs Regulation (EU) 2019/1021 and its updates. New amendments 2023/866 and 2023/1608 have been introduced to the POPs Regulation [36], and emergent contaminants and perfluoroalkyl substances (PFAS) are under observation. The EU adopted the chemical strategy for sustainability towards a toxics-free environment through new actions and revision of EU legislation. Some POPs are banned, while others are permitted but monitored, and some are regulated by the Commission working groups.

The most well-known examples are pesticides (such as DDT), dioxins, and polychlorinated biphenyls (PCBs), unintentional by-products of industrial processes and waste incineration. Dioxins are highly toxic to the reproductive system and developmentally damage the immune system, interfere with hormones, and cause cancer. In general, based on their sources, POPs can be divided into those from primary and secondary sources. Primary sources involve the direct emission of POPs into the environment during their use and production, while the secondary sources involve the later redistribution of POPs between the air, soil, and water. Primary POPs dispersed in the air can accumulate in surface soils through the action of the water and in the atmosphere, which form secondary sources of POPs that can be remobilized as secondary emissions to the atmosphere. Some organic pollutants are absorbed by plants and animals and find their way into the food chain to eventually be consumed by humans, thus endangering human health. POPs exhibit a high affinity towards biological macromolecules (nucleic acids, proteins, lipids), and they can cause genotoxicity [33].

**Figure 3:**
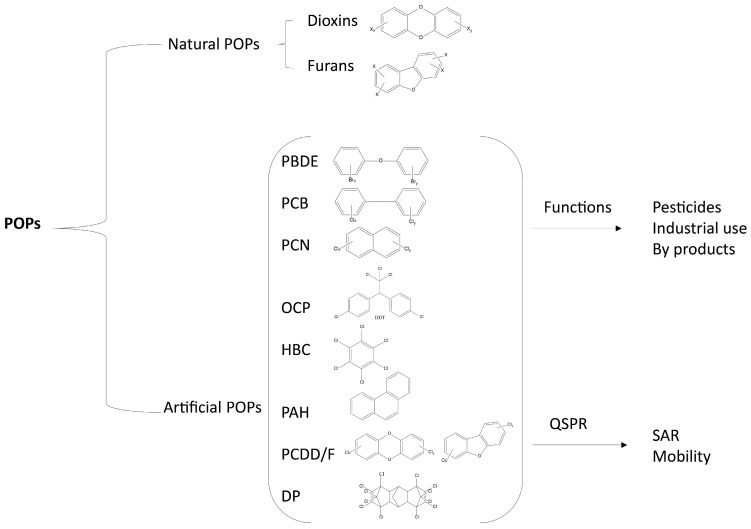
Classifications of POPs based on their origin. Artificial POPs can be classified into two categories: Function and Quantitative Structure Property Relationships (QSPRs). Classification is based on different molecular descriptors and statistical models, Structure–Activity Relationship (SAR), and Mobility.

Recently, Nagar [37] reported simple classifications of POPs based on their origin and the parent structure (Figure 3). The various classes of POPs reported by Nagar include natural and artificial compounds. The first class consists of two typologies of compounds, furans and dioxins, while artificial POPs are divided into eight classes: polybrominated diphenyl ethers (PBDEs), polychlorinated biphenyls (PCBs), polychlorinated naphthalene (PCN), organochlorine pesticides (OCPs) and polychlorinated dibenzo-p-dioxins/furans (PCDD/Fs), hexachlorobenzene (HCB), dechlorane plus (DP) and polycyclic aromatic hydrocarbons (PAHs).

Food plants, Cucurbitaceae species, can accumulate high concentrations of POPs [38] depending on genotypic variability and environmental parameters. The chlordane concentration in the edible tissue of crops was measured in contaminated soil and laboratory experiments [39]. The crops can be divided into three categories based on the concentration of chlordane: uptakers (zucchini, beets, carrots, dandelion, lettuce, potatoes, and spinach), non-uptakers (corn, pepper, and tomatoes), and intermediates (bush beans and eggplant). In chayote fruits (*Sicyos edulis* Jacq.), accumulation of high concentrations of chlordecone was reported [40].

Pesticides used as fungicides, insecticides, and herbicides are chemical compounds commonly used on food plants to control pests that may damage the crops during growth, storage, and transport. A meta-analysis of the literature data (1995–2021) on fruit pesticide residues, fruit type, pesticide type, and kind of mainland displayed a high heterogeneity of data collection elaborated with statistical analysis. Twenty-six different fruit types were examined. Fungicides are the prevalent pesticide residues in raspberry, followed by blueberry, yuza, grapefruit, plums, lemon, and strawberry. Fruits from South America present higher values. The review also presents the high heterogeneity of the data due to country richness and local legislation [41].

Among emergent contaminants, the bioaccumulation of cyclic C_6_O_4_, a perfluoroalkyl ether, a substitute of perfluorooctanoic acid (PFOA), was investigated in maize (*Zea mays* L.) and tomato (*Solanum Lycopersicum* L.). The contaminant was bioaccumulated in the maize, and higher values were observed in the roots, while lower values were observed in the aboveground tissue. In the tomato, very low uptake of the contaminants was present only in the leaves, but there was a delay in the fruit ripening and biomass [42]. Accumulation of PFAS was studied with grafted plants of tomatoes with contaminated waters and soil. Tomato accumulated C_4_–C_6_ short carbon chain PFAS, perfluoroottansulfonic acid (PFOS), and perfluorooctanoic acid (PFDA). C_8_–C_10_ longer carbon chain PFAS was not translocated in the leaves or the fruits in significant quantities, and PFOA, C_8_ carbon chain, was accumulated only in the leaves, suggesting a different mode of molecular transport along the plant that should be investigated. Grafted plants of tomatoes, with their greater vigor, accumulated more PFAS than non-grafted plants [43].

### 2.3. Other Chemical Hazards

Mycotoxins and phycotoxins are both subclasses of contaminants [3,44]. While mycotoxins are produced by fungi or molds, phycotoxins are derived from algae. Mycotoxin infestations may occur in several cereals, while phycotoxins are produced by algae and can accumulate in edible algae and aquatic organisms. Mycotoxins are secondary metabolites that, depending on the dosages, can cause acute toxicity and diseases, such as cancer, immunity suppression, and neural tube defects in humans, livestock animals, and pets. Many countries have established regulations to limit exposure to mycotoxins to protect humans and animals from health risks [3]. The main prevalent mycotoxins in cereal crops are Aflatoxins (AFB1, B2, G1, and G2), ochratoxin A (OTA), citrinin, patulin, trichothecenes, T2-toxin ((T2) and HT2)), fumonisins (FB1, FB2, and FB3), and zearalenone (ZEA). These toxins are produced primarily by the fungi genera *Aspergillus*, *Fusarium*, *Penicillium*, and *Alternaria*. The route of human exposure to mycotoxins is usually oral [44]. Mycotoxin infestations depend on environmental factors, including temperature and humidity. Good Agricultural Practices (GAPs) and chemical and biological control during cultivation and storage may reduce fungal infestation and mycotoxin production in cereal grains. Phycotoxins are small to medium molecules (∼300–3500 Da) that belong to many different groups of chemical compounds with different toxic mechanisms of action, and they can be present in algae-based foods (soups, sauces, pasta) [45].

Microplastics (MPs) are defined according to the US NOAA as fragments of plastic with a length of less than 5 mm; due to their slow degradation in soil, they are classified as pollutants in ecosystems [46]. In the last few years, the papers on MPs have increased exponentially, especially in aquatic environments. The sources of MPs and their dispersion in the environment are clearly shown in a recent review [47]. Interest in MPs’ effects in the soil shows growth in the recent literature, and information regarding the relationship between the soil and the crop system is still scarce, but some interesting results are rising. MPs are vectors of contaminants (antibiotics, metals, polycyclic aromatic hydrocarbons, POPs) that can bioaccumulate through the food chain and propagate their toxicity through all organisms involved.

In laboratory experiments, the soybean seeds (*Glycine max* L.) were utilized to understand the role of MPs with different chemical compositions (polyvinyl chloride (PVC), polyethene (PE), and polystyrene (PS)) in the soil and the plant. MPs influenced plant growth with direct plastic toxicity (nanoparticles), with a toxicity of the pollutants present in the plastic (metals, polycyclic aromatic hydrocarbons), thus modifying the community structure of the soil (in particular, PVC), altering the carbon flow through the soil–plant system, and adversely influencing many carbon-dependent soil functions and ecosystem services [48].

MPs and nanoplastics (NPs) are absorbed by plants through the roots and transported to the upper parts of the leaves, stems, fruits, flowers, and seeds. Or, they can enter through the leaves and be transported to the roots through atmospheric deposition [49]. MPs affect the oxidative stress and antioxidant defense system and determine cytogenotoxicity in plants, thus altering DNA synthesis and causing chromosomal damage. Phytohormones and other plant growth regulators (silicon, nitric oxide, calcium) can display synergistic or antagonistic effects and reduce phytotoxicity in plants. Experiments to reduce the MPs in the soil take advantage of the beneficial effects of biochar in plant growth regulators.

## 3. Conclusions

In the analyzed literature, some relevant research aspects are emerging. Safe crop production can be achieved through co-cropping, a promising approach from older agronomic techniques. Co-cropping has been suggested to reduce the metal intake by increasing the phytoextraction through the combination of different plant species [50]. Among old agronomic practices that show new opportunities, the rotation between wheat and legume crops increases soil water infiltration and increases soil surface roughness, thus reducing water runoff and soil water storage, a useful tool within climate changes [51].

The bioaccumulation of contaminants depends on environmental conditions and plant genetics. Some cultivars have low levels of accumulation, while others have high levels, and the selection of cultivars most suitable for contaminated areas should be improved and monitored to produce safe foods. Metals and POPs could show different responses that should be investigated more deeply [25,43]. Organic contaminants in the wastewater used for irrigation are degraded by biofilters (consortium of microorganisms), which biotransform organic compounds and generate biomass. Heavy metals are not biodegraded, but biofilters generate less soluble form of metals that can be adsorbed or co-precipitated and provide cost-effective solutions for contaminated water treatment.

Studies are required on the fate of micro- and nanoparticles and their effect on different plant species and food safety [50]. Nanomaterials, like molybdenum disulphide in the monolayer sheet form, are used in different sectors, leading to the inevitable release of nanomaterials in the environment. Alteration of rice roots in laboratory studies and omics techniques will provide novel insights into the ecological risk of nanomaterials to drive the application and management of molybdenum disulphide in the environment [28]. Studies on plant growth regulators and plant hormones could be performed to reduce phytotoxicity in food plants.

The mitigation strategies will depend on the types of vegetables and food crops. Biochar, biofilter, microbes with biochar, fertilizers, and co-cropping significantly reduce Pb bioavailability in vegetables. Soil amendments can reduce the metals’ uptake [52] and emergent contaminants [53], and nanoscience and transgenic plants offer possibilities for developing environmentally safe, cost-effective, and sustainable remediation. However, there is no single technique that can efficiently clean the soil from heavy metal contamination, and it is necessary to adopt integrated approaches [50].

Several studies correlate contaminants with the food chain, but few studies correlate the chemical composition of particulate matter (PM), the water, the soil, and the uptake of vegetables with integrated techniques and omics approaches for deeper knowledge of food plants’ bioaccumulation. Biomonitoring studies indicate that plant species with a high surface-to-volume ratio accumulate more POPs from the air than species with compact leaves, and the difference in leaf morphology and physiology affects the accumulation [4]. These results can help to draw new environmental strategies to lower the atmosphere’s pollution and produce safe foods.

To address and mitigate the risks of contamination inside food plants, various measures can be taken, including regular monitoring of the soil, water, and air quality, implementing proper waste management practices, using organic farming methods, employing integrated pest management strategies to reduce POPs, improving environmental protection laws, and increasing new technological skills to identify emerging contaminants in the environment. Promoting sustainable agriculture practices can help reduce the overall environmental impact and enhance the resilience of food plants.

The relationship between food plants and environmental contamination is complex, and the literature published over the last few years shows the increasing interest in safe foods. Organizations (FAO, WHO, UNECE) and researchers have started to pay attention not only to the production of new foods but also to the production of safe foods from a microbiological point of view that are also free from contaminants. The concept of One Health is not simple. The soil, water, and atmosphere are closely linked, and the plants and their products that we and other living beings feed on comprise a single system. The high specialization in research sectors makes the transversal exchange of skills difficult, but analyzing recent literature highlights demonstrates a shift in research interests, which will certainly produce interesting and useful results in a short time.

## Data Availability

Not applicable.
